# Alcohol intake exacerbates experimental autoimmune prostatitis through activating PI3K/AKT/mTOR pathway-mediated Th1 differentiation

**DOI:** 10.3389/fimmu.2024.1512456

**Published:** 2025-01-13

**Authors:** Shun Xu, Jing Chen, Shaoyu Yue, Yifan Zhang, Shengyu Zhao, Yongtao Hu, Cheng Zhang, Wenrui Guan, Li Zhang, Ligang Zhang, Chaozhao Liang

**Affiliations:** ^1^ Department of Urology, The First Affiliated Hospital of Anhui Medical University, Anhui Medical University, Hefei, Anhui, China; ^2^ Institute of Urology, Anhui Medical University, Hefei, Anhui, China; ^3^ Anhui Province Key Laboratory of Urological and Andrological Diseases Research and Medical Transformation, Anhui Medical University, Hefei, Anhui, China

**Keywords:** alcohol, CP/CPPS, PI3K/AKT/mTOR pathway, Th1 cell, mouse model

## Abstract

**Background:**

Epidemiological investigations have revealed a significant association between alcohol consumption and chronic prostatitis/chronic pelvic pain syndrome (CP/CPPS). Nevertheless, the potential mechanisms are still inadequately revealed. This research aimed to investigate the impact of alcohol on CP/CPPS using an animal model and to elucidate the underlying mechanisms.

**Methods:**

We first established the widely used animal model for CP/CPPS, experimental autoimmune prostatitis (EAP). During the induction of EAP, mice were fed with alcohol or control diet. The HE staining, ELISA, and behavioral experiments were employed to assess the severity of inflammation in EAP mice and EAP-alcohol mice. Patients with a history of chronic alcohol consumption were also included to evaluate the effects of chronic alcohol consumption on CP/CPPS. Subsequently, proteomic analysis, flow cytometry, immunofluorescence, Western blotting, and immunohistochemistry were utilized to investigate the underlying mechanism involved both *in vivo* and *in vitro*.

**Results:**

HE staining, ELISA, and behavioral experiments showed that alcohol exacerbated the severity of EAP in mice and patients. Proteomic and KEGG pathway analyses showed that abnormal Th1 differentiation and PI3K/AKT/mTOR pathway were significantly enriched. Subsequent mechanistic research showed that alcohol significantly activated PI3K/AKT/mTOR pathway and increased the Th1 cell differentiation both *in vivo* and *in vitro*. In contrast, PI3K inhibitor LY294002 and shRNA-PI3K plasmid inhibited PI3K/AKT/mTOR pathway activation, reduced Th1 cell differentiation, and alleviated EAP inflammation severity, respectively.

**Conclusion:**

Our study is the first to demonstrate that alcohol intake promotes Th1 cell differentiation and exacerbates EAP by activating the PI3K/AKT/mTOR pathway. Additionally, the role of LY294002 in inhibiting PI3K/AKT/mTOR pathway to relieve EAP suggests that it can serve as a promising therapeutic target for CP/CPPS.

## 1 Introduction

The chronic prostatitis/chronic pelvic pain syndrome (CP/CPPS) is a typical urogenital disease affecting young males, accounting for over 25% of urology outpatients. In China, epidemiological studies indicated that the occurrence of CP/CPPS symptoms is about 8.4% of the population. Additionally, about 50% of all men suffer CP/CPPS symptoms at some age stage (1). CP/CPPS symptoms are often varied, with feature of pain and discomfort in the testis, scrotum, and perineum, bladder irritation symptoms (frequency, urgency, dysuria), abnormal urodynamic performance (2), and an increased risk of male infertility and sexual dysfunction. However, the exact pathogenesis of prostatitis remains unknown.

Alcohol consumption is widespread globally. Consequently, alcohol-related disorders are highly common worldwide (3, 4). Alcohol consumption affects nearly all organs and is intricately linked to various inflammatory diseases, like hepatitis (5), pancreatitis, and atherosclerosis (6, 7). Epidemiological researches revealed a strong relation in alcohol intake and CP/CPPS (1). Individuals with drinking habits exhibit a higher incidence of CP/CPPS and more severe symptoms (8). Alcohol is known to exacerbate prostate tissue inflammation, pelvic pain symptoms, and the concentrations of associated inflammatory cytokines in experimental autoimmune prostatitis (EAP) mice (9). Additionally, we observed that during alcohol-induced exacerbation of EAP, IFN-γ levels were significantly elevated, suggesting that Th1 cells may play a regulatory role in alcohol-induced EAP.

Upon activation, initial CD4+ T cells differentiate into various other cells, like Th1, Th2, Th17,depending on the cytokines they produce (10). Th1 cells have a strong pro-inflammatory effect (11), primarily secreting cytokines IFN-γ and IL-22, which activate neutrophils. A recent study on apical periodontitis revealed that IFN-γ levels in the apical periodontitis group were significantly upregulated compared to controls. This suggests that Th1 cells may modulate inflammation (12). Jing et al. discovered that hydrogen sulfide dysregulates the Th1/Th2 cell balance, caused higher expression of pro-inflammatory cytokines, which interferes with the insulin axis and leads to glucose metabolism disorders during the inflammatory response in chicken skeletal muscle (13). Given this evidence, it indicated that Th1 cells are strong contributors to the development and progression of inflammation.

The PI3K/AKT/mTOR pathway is frequently activated during inflammation. It works alongside other pathways to modulate the pathological process of many kinds of inflammatory diseases, such as hepatitis (14), airway inflammation in COPD (15), and osteoarthritis (16). Interestingly, PI3K/AKT/mTOR pathway is markedly activated in EAP (17). Multiple studies have indicated that alcohol may also activate the relevant axis (18, 19).

Combined with literature research and preliminary experiments, we demonstrated that alcohol activates PI3K/AKT/mTOR signaling axis. Moreover, the Th1 cell ratio strongly modulates EAP. However, the molecular mechanism underlying this process is currently unknown. The non-obese diabetic (NOD) murine EAP model was generated through immunization with CFA (20). Herein, we explored the function of CD4+IFN-γ+ Th1 cells in pain and inflammatory manifestations in EAP mice. Besides, we discovered that the alcohol-mediated effects on Th1 cells were mediated via PI3K/AKT/mTOR pathway. The above results highlight potentially potent targets for CP/CPPS therapy.

## 2 Materials and methods

### 2.1 Mice and antigens

Six- to eight-week-old NOD (NOD/LtJ) mice were acquired from Jiangsu GemPharmatech Co., Ltd. (Nanjing, China). The Animal Center provided a specific pathogen-free environment, where all the mice were housed. All protocols involving mice received ethical approval from the Ethic Committee of Anhui Medical University(No. LLSC20210270).

Prostate glands from Wistar rats (Beijing Vital Company) were collected as experimental samples. Samples from 30 rats were processed by initial homogenization in 0.01 M PBS (pH 7.2) using Super Homogenizer (Bertin, France). Homogenization was performed with protease inhibitors. Afterward, samples were centrifuged for 30 minutes at 10,000g, 4°C. The BCA detection Kit (Beyotime, China) was applied to quantify supernatants protein, also called prostate antigens (PAgs), and were preserved at −80°C. Next, prostate antigens (PAgs) and complete Freund’s adjuvant (CFA, Sigma-Aldrich) were mixed in a 1:1 ratio and fully emulsified using ultrasonic processing to form a suspension.

### 2.2 Mouse model of EAP

PAgs emulsified in CFA (150μL) were intradermally administered to NOD mice (300μg/mouse, EAP mice), while PBS was administered to control mice at their tail base, hind footpad, and lower back as previously outlined (21). Following a previous immunization schedule (22, 23), mice received these injections on days 0, 28, and were sacrificed on day 42.

### 2.2.1 Alcohol feeding protocols

32 days post-PAgs or PBS treatments, two groups of mice—one control‐alcohol (CA) group and one EAP‐alcohol (EA) group—were persistently fed with alcohol. This was achieved by ad libitum oral feeding of the Lieber‐DeCarli alcohol diet for 10 days, along with a single gavage of 35% alcohol (5 g alcohol per kg). Additionally, two groups of mice (control-vehicle, CV, EAP‐vehicle, EV) were given a control diet. The alcohol intake procedure has been previously described (24).

### 2.2.2 Treatment with LY294002

EAP mice in the EAP-LY294002 (E-LY) and EAP-alcohol-LY294002 (EA-LY) groups were administered daily intraperitoneal (IP) injections of 5mg/kg LY294002 (Selleck, S1105) from days 28 to 42 (25). The EAP-vehicle (EV) and EAP-alcohol (EA) mice received intraperitoneal PBS injections at the same time intervals.

### 2.3 Patients

This research was performed in line with the Declaration of Helsinki and was approval by the Ethic Committee of the First Affiliated Hospital Anhui Medical University (PJ2024-03-37). Prostate tissue specimens were collected from 60 benign prostatic hyperplasia patients who underwent laser enucleation treatment. Inclusion criteria: 1. Diagnosis of benign prostatic hyperplasia (BPH); 2. Presence of surgical indications; 3. Provision of informed consent. Exclusion criteria: 1. Presence of malignant tumors; 2. History of urinary system surgery; 3. Presence of severe infectious diseases; 4. Presence of coagulation disorders. HE staining was applied to assess the prostate tissues inflammation. The inflammation severity of these prostate tissues was graded based on clinical pathology reports. Patients were grouped according to the history of chronic alcohol consumption.

### 2.4 Histopathological evaluation

We conducted H&E staining of the prostate tissue of mice. Initially, the specimens were fixed for 1 d. They were sequentially dehydrated in graded alcohol and xylene series. Subsequently, the specimens were embedded and sliced into approximately 5-μm sections. These sections underwent H&E staining and were observed via microscope. Histopathological assessments used a point-counting system to evaluate inflammation severity, as previously described (9). Cellular alterations were graded on a four-point scale (0–3): 0 for no inflammation, 1 for mild inflammation characterized by perivascular cuffing, 2 for moderate inflammation with medium mononuclear cells, while 3 for severe inflammation with significant perivascular cuffing, abundant mononuclear parenchymal cells, and hemorrhage.

### 2.5 Behavioral testing

Cutaneous allodynia was assessed 42 days following the initial injection. An isolated transparent plastic chamber was used to conduct 24 evaluations, based on a previously established protocol (9, 17). Three situations were considered positive outcomes of filament stimulation. These included: (1) intense abdominal retraction, (2) instantaneous scratching or licking of the filament stimulation site, (3) jumping. The response rate frequency was determined based on the ratio of positive outcomes (e.g., 10 applications resulting in 3 outcomes = 30%).

### 2.6 ELISA

Cytokine contents were assessed using mouse plasma from EAP mice injected subcutaneously with PAgs and an ELISA, following kit directions (IFN-γ: Elabscience Biotechnology Co. Ltd., Wuhan, China; TNF-α: E-EL-M3063, Elabscience Biotechnology Co. Ltd., Wuhan, China; IL-4: E-EL-M0043c, Elabscience Biotechnology Co. Ltd., Wuhan, China; IL-10: E-EL-M0046c, Elabscience Biotechnology Company., Wuhan, China). The assay linear ranges were as follows: 15.63-1000 pg/mL (IL-10 and IFN-γ), 31.25-2000 pg/mL (IL-4), 7.81-500 pg/mL (TNF-α).

### 2.7 Western blotting analysis

Tissues and cell lysis were performed in RIPA buffer for 30 minutes at 4°C before adding SDS loading buffer. Protein separation was performed using SDS-PAGE gel (Bio-Rad) and transferred onto PVDF membranes. The membranes were blocked with primary antibodies against PI3K (1:1000; CST,#42495), p-PI3K (1:1000; CST, #17366), AKT,p-AKT,mTOR, p-mTOR, and GAPDH (1:3000; Affinity, AF7021) at 4°C. This was followed by antibodies marked by HRP (1:5000; goat anti-rabbit) for 1 hour. Protein visualization and quantification were performed using chemiluminescence with a ChemiScope and ImageJ (National Institutes of Health).

### 2.8 Immunohistochemistry evaluations

The prostate specimen was fixed for 1 day, subsequently paraffin embedding and sliced into 5-μm sections. The paraffin slices were dewaxed using xylene. After rehydration, antigens were retrieved by microwaving the slices for 15 minutes at 95°C in 0.01 M citric acid buffer. The sections were processed with 3% H_2_O_2_ for 10 minutes (SP 9000; Beijing Zhongshan Company) at 25°C. Next, it was washed with PBS (pH 7.4) before blocking in 10% BSA. They were then cultured for 14h at 4°C with primary antibodies ([1:200] p-PI3K, [1:200] p-AKT, and [1:500] p-mTOR). Subsequently, it was washed with PBS and cultured with goat anti-mice IgG (1:200) for 30 minutes at 37°C, followed by three PBS rinses and treatment with horseradish peroxidase-labeled streptavidin at 37°C for 30 minutes. Following additional PBS rinses, immunoreactivity was assessed using a Diaminobenzidine Staining Kit.

### 2.9 Immunofluorescence (IF)

Paraffin sections were successively dewaxed, rehydrated, antigen repaired, and endogenous peroxidase activity quenched. BSA was then added and blocked for 30 minutes. The blocking solution was removed, and the specific primary antibodies were added. The slides were placed flat in a humidified box and incubated for 14h at 4°C. The rabbit anti-CD4 polyclonal antibody (YT0762, Immunoway) and rabbit anti-IFN-γ polyclonal antibody (YT2279, Immunoway) was applied in the experiment. Next, the slides were treated with fluorescent secondary antibodies, covered with an incubation box. The slides were counterstained with DAPI for nuclei staining and then sealed with sealing agent. A fluorescence microscope was applied for photography.

### 2.10 Flow cytometry and ICS

Lymphocytes were extracted from murine spleens and stained with fluorescence-labeled anti-CD4 (BD Pharmingen, 553650). To conduct ICS, cells from murine NOD-derived spleen samples were treated in 1640 medium (Gibco, USA) for 4 hours with ionomycin (MultiSciences, China), and monensin.

Following a 60-minute surface marker CD4 (BD Pharmingen, 553650) stain at 4°C, Fix-Perm intracellular buffers were utilized for cell fixation and permeabilization. Intracellular cytokine antibodies (anti-IFN-γ, BD Pharmingen, 554412) were applied to stain cytokines for 1h at 4°C. A flow cytometer (Beckman Coulter, Brea, CA) assessed the stained cells, and data analysis was conducted using CytExpert Software.

### 2.11 Cell isolation and *in vitro* differentiation of naive CD4+ T cells

The unlabeled cells were collected via LS Column in line with relevant Isolation Kit (Miltenyi Biotec). IL-2 (10 ng/mL;CK24), IL-12 (10 ng/mL; Novoprotein, CM39), and anti-IL-4 (BE0045) were used for the generation of Th1 cells. To investigate the effect of alcohol on Th1 cell differentiation, 100 mM alcohol was added to the 1640 medium with 10% FBS (26, 27). For the intervention group, naive CD4+ T cells were transfected with shRNA-PI3K plasmid or NC plasmid (Genechem, China). After 4 days of culture, cells were harvested and relevant experiments were performed.

### 2.12 Short hairpin RNA (shRNA) plasmids inhibit the expression of target genes

The knockdown plasmid, purchased from Shanghai Genechem Company (Contract Number: GIEE0429289), was used to inhibit the PI3K gene with short hairpin RNA (shRNA). The shRNA-PI3K plasmid sequence used was: 5’-GATCCCGTACGAGACGCATCTACTAAACTCGAGTTTAGTAGATGCGTCTCGTACTTTTTGGAT-3’. The sorted naive CD4+ T cells were transfected for 1h, and the medium was then changed, followed by the corresponding stimulation and differentiation operations.

### 2.13 Proteomic sequencing and bioinformatics analysis

Tissue samples were collected from four groups of NOD mice (CV, CA, EV, EA), and proteins were extracted via the RIPA lysis buffer. The proteins were digested with trypsin and treated via LC-MS/MS. The obtained data were applied to identify proteins. The identified proteins were annotated using the GO and KEGG databases. Enrichment analysis was conducted. The obviously enriched pathways and biological processes were identified with a threshold of P < 0.05.

### 2.14 Statistical analysis

Data were expressed as mean ± SD and were compared via independent t-tests or ANOVA. Statistical difference was judged by *P < 0.05. In the figures, *, **, ***, and **** represent statistical significance levels of P < 0.05, P < 0.01, P < 0.001, P < 0.0001, respectively.

## 3 Results

### 3.1 Establishment of the EAP model and Th1 abnormalities

**Figure 1:**
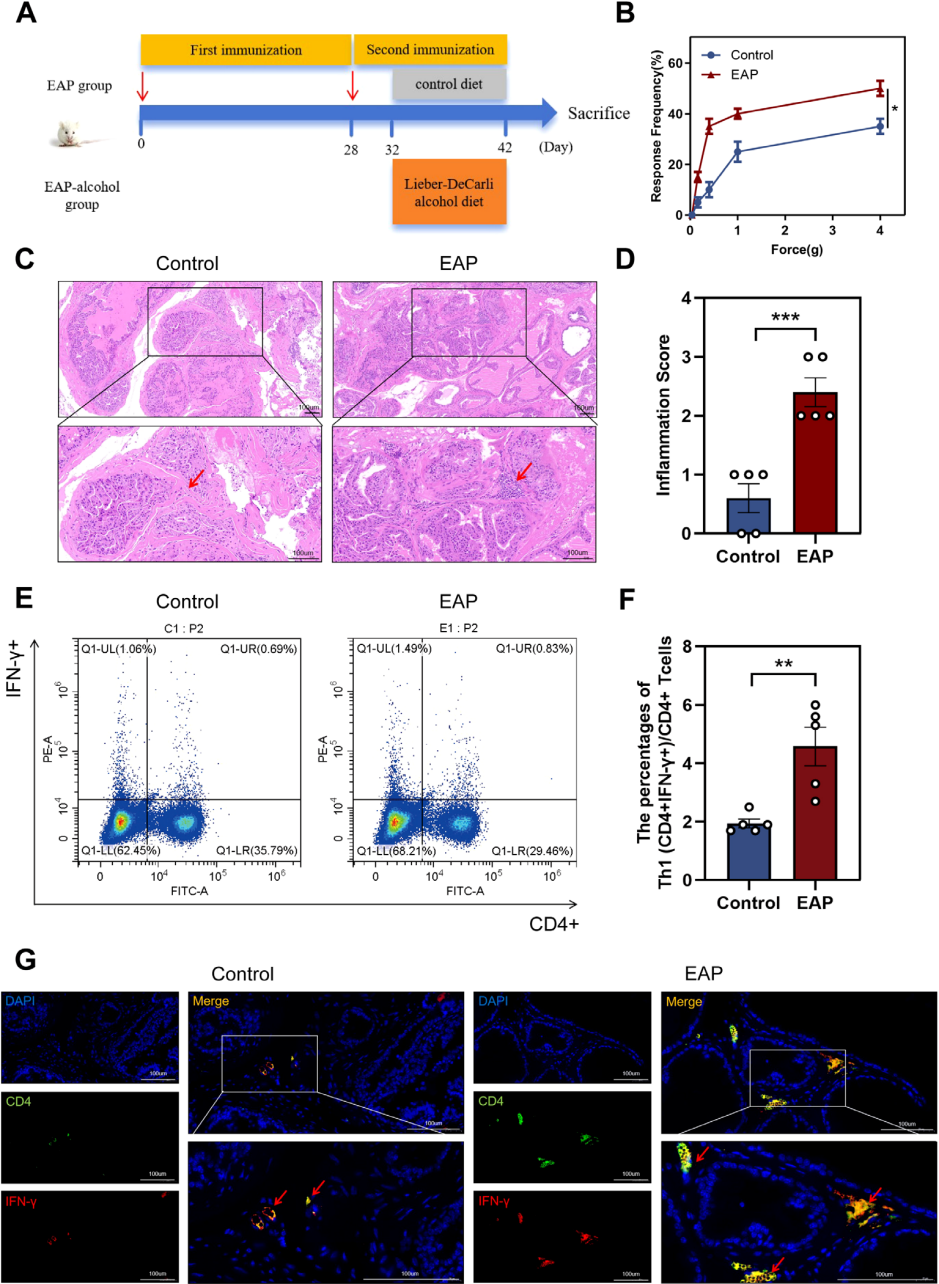
Induction of EAP model and abnormalities in the Proportion of Th1 in EAP mice. **(A)** Flow chart of EAP mice induction. **(B)** EAP induced pelvic pain as assessed by tactile allodynia von Frey testing. **(C)** Representative HE staining images of prostate tissue sections of control and EAP group. **(D)** Inflammation score of prostate tissue in control and EAP group. **(E)** Representative pictures of flow cytometric staining for Th1 cells in the splenic lymphocytes from control and EAP mice. **(F)** Flow cytometric analysis of the proportion of Th1 cells in control and EAP mice. **(G)** Representative pictures of immunofluorescent staining for Th1 cells in the splenic lymphocytes from control and EAP mice. NS, no significance, *P < 0.05, **P < 0.01, ***P < 0.001; EAP, experimental autoimmune prostatitis; HE, hematoxylin-eosin.

The EAP model was established (**Figure 1A**). The EAP model was assessed using histological analysis and pelvic pain assessment in this study. EAP mice exhibited significantly higher tactile allodynia responses to forces of 0.4 g, 1.0 g, 4.0 g than control mice (*P* < 0.05, **Figure 1B**). EAP mice exhibited pathological alterations such as stromal mononuclear cellular invasion, edema, chronic tissue disorders (**Figure 1C**). HE scores for EAP and control mice are depicted in **Figure 1D** (*P <* 0.001).

Next, we assessed the ratio of Th1 cells among splenic lymphocytes from EAP and control mice. Our data revealed a significantly higher concentration of Th1 (CD4+IFN-γ+) cells in EAP mice than that of controls (*P* < 0.01, **Figure 1E, F**). This finding was further confirmed by immunofluorescence staining results (**Figure 1G**), demonstrating an increased ratio of Th1 cells in EAP mice.

### 3.2 Alcohol exacerbated the severity inflammation

**Figure 2:**
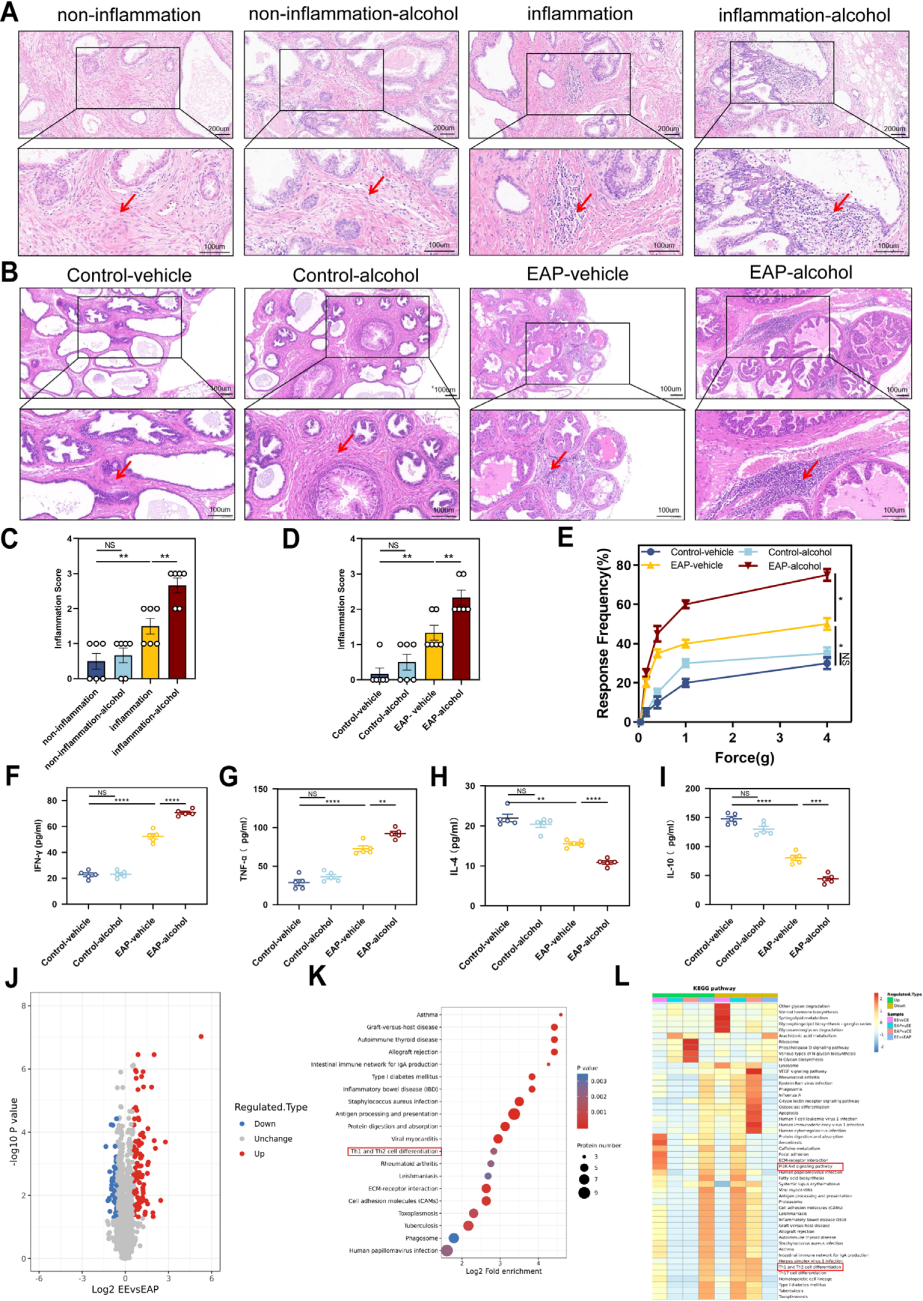
Alcohol worsened EAP mice and Benign prostatic hyperplasia patients degree, and Proteomic features of EAP-alcohol and EAP mice. **(A)** Representative HE staining images of prostate tissue sections of non-inflammation, non-inflammation-alcohol, inflammation, and inflammation-alcohol group. **(B)** Representative HE staining images of prostate tissue sections of control-vehicle, control-alcohol, EAP-vehicle, and EAP-alcohol group. **(C)** Inflammation score of prostate tissue in non-inflammation, non-inflammation-alcohol, inflammation, and inflammation-alcohol group. **(D)** Inflammation score of prostate tissue in control-vehicle, control-alcohol, EAP-vehicle, and EAP-alcohol group. **(E)** Pelvic pain as assessed by tactile allodynia von Frey testing. **(F–I)** Concentration determination of serum IFN-γ, TNF‐α, IL-4, and IL-10 by ELISA in control-vehicle, control-alcohol, EAP-vehicle, and EAP-alcohol group. **(J)** Volcano plot analyses of protein expression levels in EAP-alcohol and EAP-vehicle mice prostate tissue. The red origin represents the significantly up-regulated protein, the blue origin represents the significantly down-regulated protein, and the gray dot represents the insignificant protein. **(K)** Selected examples of KEGG pathway enrichment. Disturbances in Th1 and Th2 cells are highlighted in red. **(L)** Hierarchical clustering heatmap of protein expression levels in EAP-alcohol and EAP-vehicle mice. Disturbances in PI3K/AKT network are highlighted in red. NS, no significance, *P < 0.05, **P < 0.01, ***P < 0.001, ****P < 0.0001; EAP, experimental autoimmune prostatitis; HE, hematoxylin-eosin.

H&E staining indicated that patients with benign prostatic hyperplasia who have a history of drinking exhibit significantly higher levels of inflammation in their prostate tissue compared to those with no experience of drinking (*P* < 0.01, **Figure 2A, C**). This finding aligns with the outcomes observed in the animal experiments. EA mice showed more severe prostate tissue inflammation than the EV mice (*P* < 0.01, **Figure 2B, D**). Severe inflammation was pronounced in the stromal tissue with a significant infiltration of mononuclear cells, as well as fibrosis and hemorrhage. In contrast, CV and CA mice showed no clear signs of inflammatory cell infiltration (**Figure 2B, D**). EA mice exhibited significantly higher tactile allodynia responses to forces of 0.4 g, 1.0 g, 4.0 g than EV mice (*P* < 0.05, **Figure 2E**).

The profile of specific inflammatory cytokines was assessed in the plasma of immunized mice using ELISA. The IFN‐γ and TNF‐α (*P* < 0.01) were significantly elevated in EA mice than that in EV mice (**Figure 2F, G**). The cytokines IL‐4 (*P* < 0.0001) and IL‐10 (*P* < 0.001) showed significant downregulation compared to the controls (**Figure 2H, I**). This evidence suggests that alcohol significantly relieve the EAP in mice and benign prostatic hyperplasia in patients.

### 3.3 Impact of alcohol on prostatitis: insights from proteomic analysis

As previously noted, alcohol significantly influences prostatitis(9). Therefore, we conducted proteomic analysis to clarify the potential mechanism of action in EA mice. Analysis of differential protein expression profiles in alcohol-induced experimental autoimmune prostatitis mice showed that EA and EV mice exhibited 152 differences in protein expression, with 100 proteins up-regulated (**Figure 2J**).

Proteomic and KEGG pathway analyses showed that multiple pathways were enriched. Specifically, the pathway related to T lymphocyte differentiation strongly influenced the development and progression of EAP. Based on analyses and identification, abnormal Th1 differentiation was chosen as the focus of our research (**Figure 2K**).

Sequencing also revealed that the PI3K/AKT/mTOR axis was activated after alcohol treatment (**Figure 2L**). The results strongly indicated that alcohol-induced activation of that axis mediated Th1 differentiation, playing a crucial role in EAP progression. After conducting a thorough literature review, we identified that pathway as the focus of our research. Taken together, these findings highlighted significant differences between EA and EV mice, providing insights into the role of alcohol in regulating EAP.

### 3.4 Alcohol increases Th1 cell differentiation and activates the PI3K/AKT/mTOR pathway

**Figure 3:**
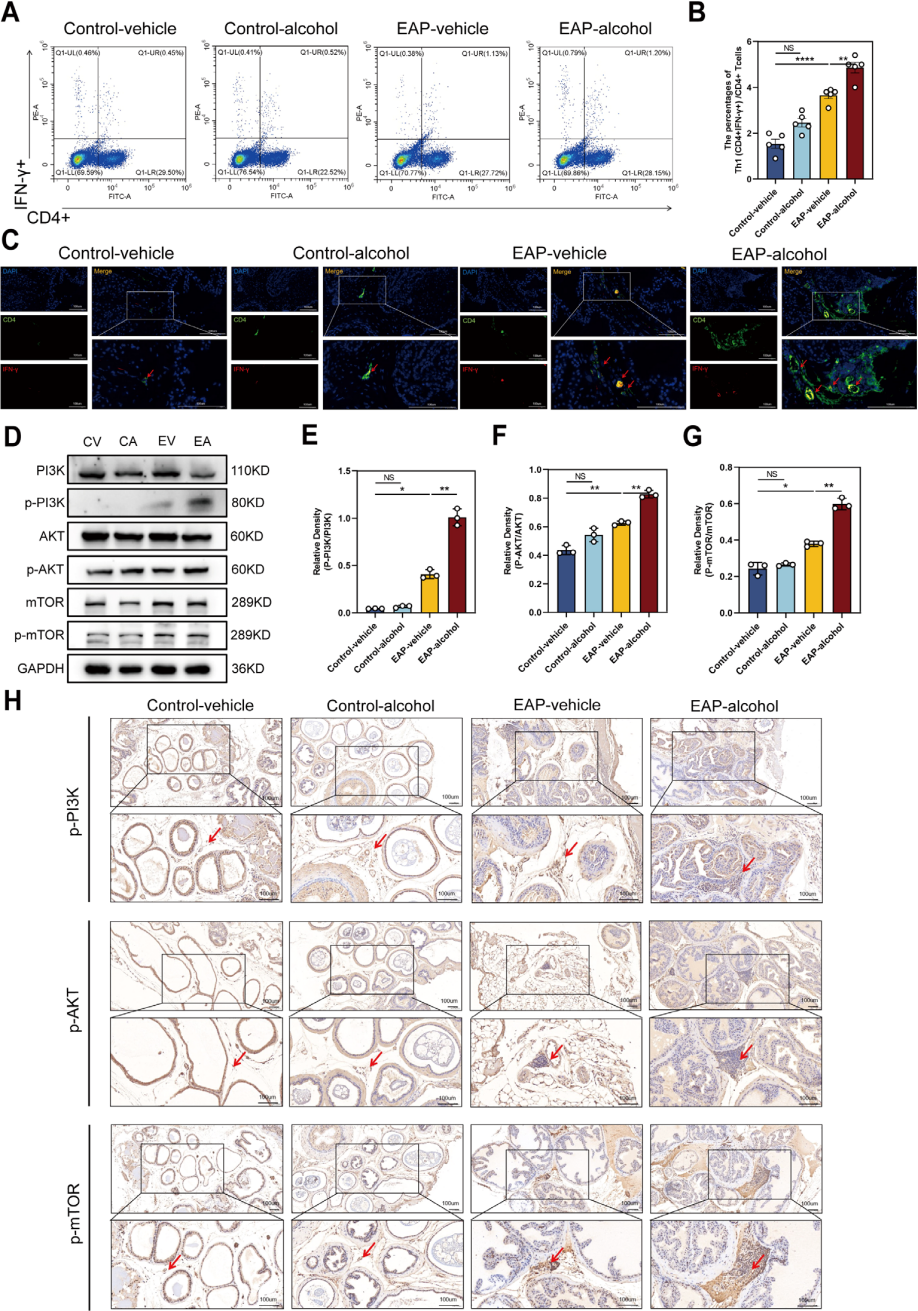
The effect of alcohol on EAP mice/patients causes an increase in the Th1 cell differentiation ratio, and activates the PI3K/AKT/mTOR pathway. **(A)** Representative pictures of flow cytometric staining for Th1 cells in the splenic lymphocytes from control-vehicle, control-alcohol, EAP-vehicle, and EAP-alcohol group. **(B)** Flow cytometric analysis of the proportion of Th1 cells in control-vehicle, control-alcohol, EAP-vehicle, and EAP-alcohol group. **(C)** Representative pictures of immunofluorescent staining for Th1 cells of prostate tissue sections from control-vehicle, control-alcohol, EAP-vehicle, and EAP-alcohol group. **(D)** The expressions of PI3K, phospho‐PI3K, AKT, phospho-AKT, mTOR, and phospho-mTOR in prostate were detected by Western blotting analysis for the CV, CA, EV, EA. **(E)** Relative density analysis of PI3K/phospho‐PI3K. **(F)** Relative density analysis of AKT/phospho-AKT. **(G)** Relative density analysis of mTOR/phospho-mTOR. **(H)** The activation of phospho‐PI3K, phospho-AKT, and phospho-mTOR showed by Immunohistochemistry Assays for control-vehicle, control-alcohol, EAP-vehicle, and EAP-alcohol group. NS, no significance, *P < 0.05, **P < 0.01, ****P < 0.0001; EAP, experimental autoimmune prostatitis.

Following alcohol exposure, we assessed the number and proportion of Th1 (CD4+IFNγ+) cells in EAP mice using flow cytometry. The data indicated that alcohol exposure significantly increased Th1 cell content in EAP mice, leading to a higher Th1 cell differentiation ratio than that of the EAP-vehicle group (*P* < 0.01, **Figure 3A, B**). Immunofluorescence staining of mouse prostate tissue (**Figure 3C**) revealed that the ratio of Th1 cells was obviously higher in the EAP-alcohol group than the EAP-vehicle group.

To clarify the mechanisms of alcohol’s effects in EAP mice, we evaluated proteins related to the pathway using western blotting and immunohistochemical (IHC) analyses. In EV mice, levels of p-PI3K/PI3K, p-mTOR/mTOR were obviously enhanced. Following alcohol exposure in EAP mice, the concentrations of these inflammation-associated proteins, specifically p-PI3K/PI3K, p-AKT/AKT, p-mTOR/mTOR, were markedly enhanced, as demonstrated by western blotting (*P* < 0.01, **Figure 3D–G**) and IHC assays (**Figure 3H**). Based on this evidence, we confirmed that alcohol activates the PI3K/AKT/mTOR pathway.

### 3.5 LY294002 reduces the inflammation severity and inhibits the PI3K/AKT/mTOR pathway

**Figure 4:**
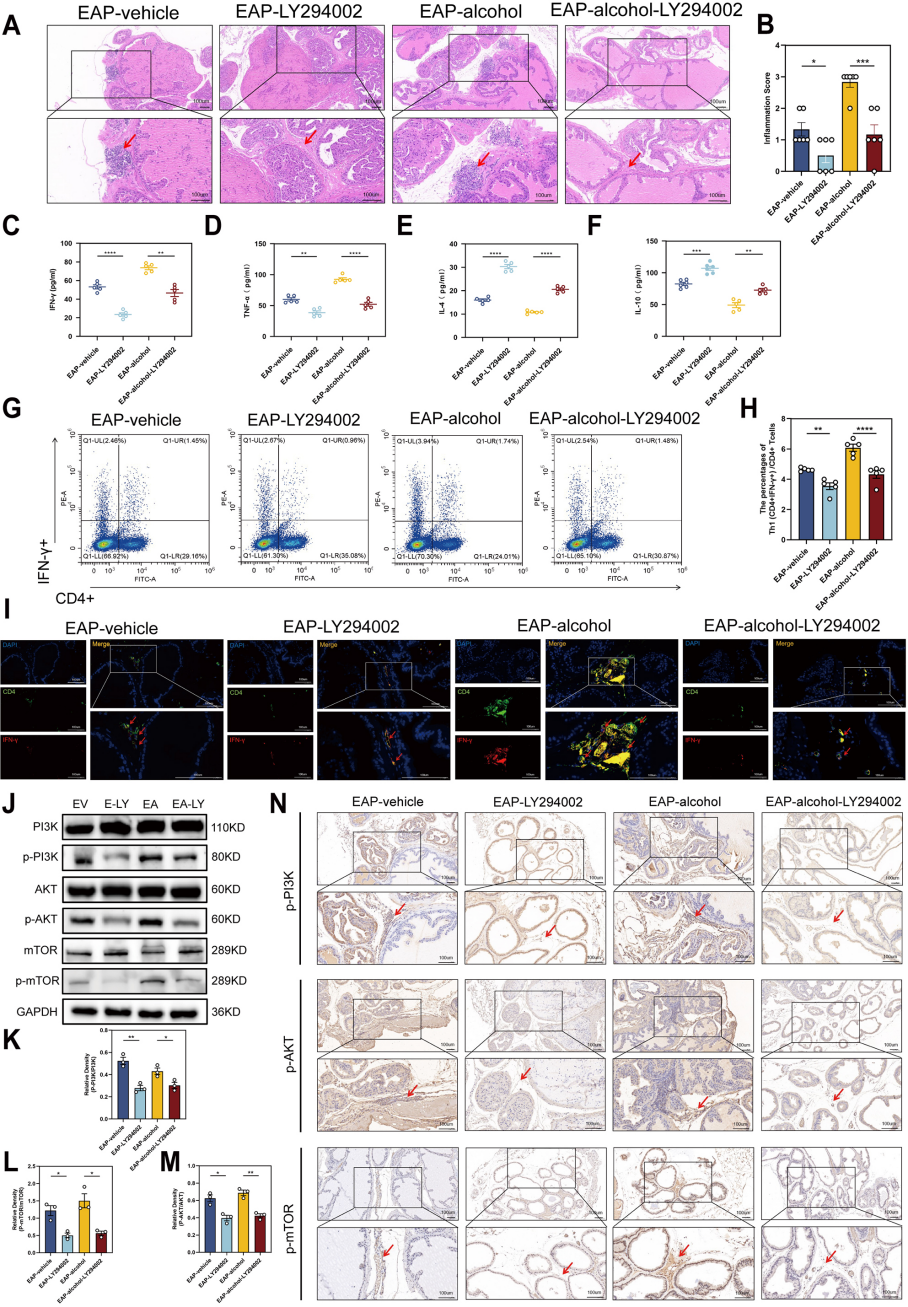
LY294002 may reduce inflammation severity, decrease the ratio of Th1 cells in EAP mouse, and inhibit the PI3K/AKT/mTOR pathway activation. **(A)** Representative HE staining images of prostate tissue sections of EAP-vehicle, EAP-LY294002, EAP-alcohol, and EAP-alcohol-LY294002 group. **(B)** Inflammation score of prostate tissue in EAP-vehicle, EAP-LY294002, EAP-alcohol, and EAP-alcohol-LY294002 group. **(C–F)** Concentration determination of serum IFN-γ, TNF‐α, IL-4, and IL-10 by ELISA in EAP-vehicle, EAP-LY294002, EAP-alcohol, and EAP-alcohol-LY294002 group. **(G)** Representative pictures of flow cytometric staining for Th1 cells in the splenic lymphocytes from EAP-vehicle, EAP-LY294002, EAP-alcohol, and EAP-alcohol-LY294002 group. **(H)** Flow cytometric analysis of the proportion of Th1 cells in EAP-vehicle, EAP-LY294002, EAP-alcohol, and EAP-alcohol-LY294002 group. **(I)** Representative pictures of immunofluorescent staining for Th1 cells of prostate tissue sections of EAP-vehicle, EAP-LY294002, EAP-alcohol, and EAP-alcohol-LY294002 group. **(J)** The expressions of PI3K, phospho‐PI3K, AKT, phospho-AKT, mTOR, and phospho-mTOR in prostate were detected by Western blotting analysis for the EV, E-LY, EA, EA-LY. **(K)** Relative density analysis of PI3K/phospho‐PI3K. **(L)** Relative density analysis of AKT/phospho-AKT. **(M)** Relative density analysis of mTOR/phospho-mTOR. **(N)** The activation of phospho‐PI3K, phospho-AKT, and phospho-mTOR showed by Immunohistochemistry Assays for EAP-vehicle, EAP-LY294002, EAP-alcohol, EAP-alcohol-LY294002. NS, no significance, *P < 0.05, **P < 0.01, ***P < 0.001, ****P < 0.0001; EAP, experimental autoimmune prostatitis; HE, hematoxylin-eosin.

Next, we explored the effect of LY294002, an inhibitor of PI3K/AKT/mTOR pathway, on the inflammation severity in mice models. After LY294002 treatment, H&E staining showed a significant reduction in perivascular and interstitial multifocal mononuclear cell invasion in EA mice (**Figure 4A**). The EAP scores of four murine groups are depicted in **Figure 4B**.

The levels of cytokines in EA-LY mice showed the following profile: IFN‐γ (*P* < 0.01) and TNF‐α (*P* < 0.0001) were significantly reduced in EA-LY mice than that of EA mice (**Figure 4C, D**), whereas cytokines IL‐4 (*P* < 0.0001) and IL‐10 (*P* < 0.01) were reversed (**Figure 4E, F**).

Moreover, we assessed the number and proportion of CD4+IFNγ+Th1 cells (*P* < 0.001, **Figure 4G, H**) in spleen lymphocytes of EAP mice using flow cytometry following intraperitoneal LY294002 administration. Immunofluorescence staining of Th1 cell-related proteins was conducted on prostate tissues from all four groups of mice (**Figure 4I**). The data showed comparable Th1 cell levels relative to EA mice, and the Th1 cell differentiation ratio was reduced in EA-LY mice. These findings suggest that LY294002 exposure has the potential to reduce inflammation in EA mice.

Subsequetly, this research explored the effects of LY294002 exposure on the PI3K/AKT/mTOR pathway and inflammation status in EA mice. Western blotting analysis result indicated that the expressions of p-PI3K/PI3K, p-AKT/AKT, p-mTOR/mTOR were obviously reduced in EA-LY mice compared to EA mice (*P* < 0.05, **Figure 4J–M**). We also evaluated PI3K/AKT/mTOR-associated proteins using IHC. The IHC data in EA-LY mice confirmed our western blotting findings, suggesting that the PI3K suppressor LY294002 prevented the PI3K/AKT/mTOR pathway (**Figure 4N**).

### 3.6 Activation of PI3K/AKT/mTOR pathway and an increase of Th1 cell differentiation *in vitro*

**Figure 5:**
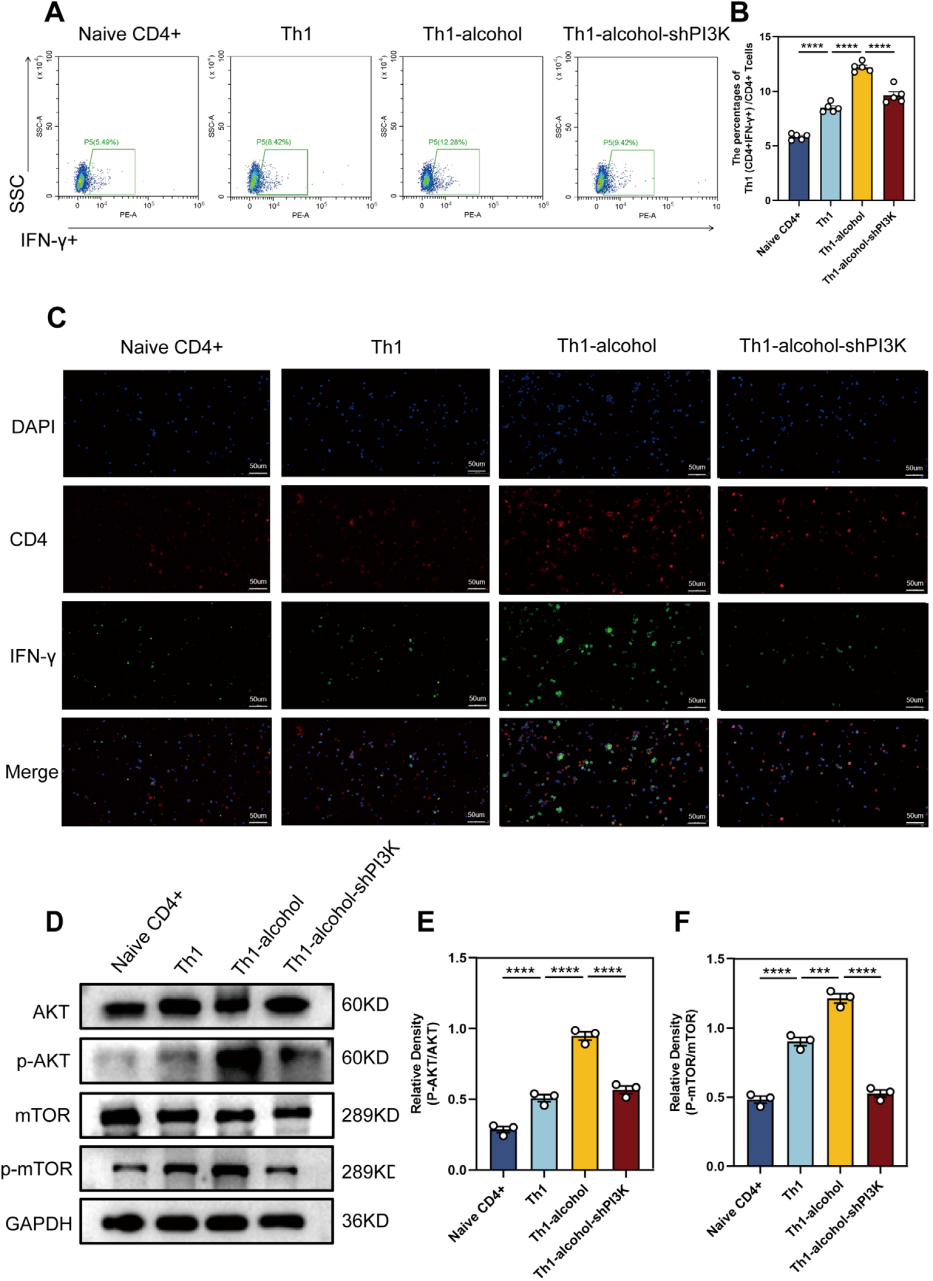
*In vitro* experiments further demonstrated increased proportion of Th1 cells and the pathway activation. **(A)** Representative pictures of flow cytometric staining for Th1 cells of Naive CD4+, Th1, Th1-alcohol, Th1-alcohol-shPI3K group. **(B)** Flow cytometric analysis of the proportion of Th1 cells in Naive CD4+, Th1, Th1-alcohol, Th1-alcohol-shPI3K group. **(C)** Representative pictures of immunofluorescent staining for Th1 cells of Naive CD4+, Th1, Th1-alcohol, Th1-alcohol-shPI3K. **(D)** The expressions of AKT, phospho-AKT, mTOR, and phospho-mTOR in lymphocytes were detected by Western blotting analysis for the Naive CD4+, Th1, Th1-alcohol, Th1-alcohol-shPI3K group. **(E)** Relative density analysis of AKT/phospho-AKT. **(F)** Relative density analysis of mTOR/phospho-mTOR. NS, no significance, ***P < 0.001, ****P < 0.0001; EAP, experimental autoimmune prostatitis; EV, EAP-vehicle, E-LY, EAP-LY294002, EA, EAP-alcohol, EA-LY, EAP-alcohol-LY294002.

To validate our findings, we explored the biological effects of alcohol on Th1 cell differentiation *in vitro*. Naive CD4+ T cells were isolated and processed. In the *in vitro* experiment, for the sorted Naive CD4 + T cells, IL-2, IL-12 and anti-IL-4 are used to stimulate the differentiation of Naive CD4 + T cells into Th1 cells. Flow cytometry (**Figure 5A, B**) results demonstrated that the Th1-alcohol group had a significantly higher proportion of Th1 cells than the Th1 group. Subsequent immunofluorescence staining results (**Figure 5C**) were consistent with these observations. Western blotting (**Figure 5D–F**) results demonstrated that alcohol significantly activates AKT/mTOR pathway. In short, Alcohol treatment significantly increased the differentiation ratio of Th1 cells and significantly activated the AKT/mTOR pathway. Mouse naive CD4+ T cells were transfected with an shRNA-PI3K plasmid to suppress PI3K expression. The inhibitory effect of the shRNA-PI3K-plasmid further corroborated this finding (**Figure 5**).

## 4 Discussion

CP/CPPS remains a prevalent global urinary system concern, and further exploration is needed to understand its pathogenesis. While the Th1/Th2 cell-mediated involvement in CP/CPPS patients is well-documented (28, 29), the regulatory mechanisms of Th1 cell differentiation remain incompletely understood.

Our research team has previously made significant progress in studying alcohol-induced prostatitis, contributing to the understanding of CP/CPPS pathogenesis (9, 17). Using preliminary proteomic sequencing, we found that EAP-alcohol mice exhibited an enhanced Th1/Th2 cell ratio and marked activation of the PI3K/AKT/mTOR axis compared to EAP mice. This observation suggests that alcohol strongly regulates Th1 cell differentiation through induction of that axis, influencing CP/CPPS pathogenesis. Our study enhances understanding of the regulatory mechanisms of Th1 cell differentiation and sheds light on the development of alcohol-induced prostatitis.

Alcohol is a well-established regulator of inflammation, significantly increasing production of pro-inflammatory cytokines like TNF‐α, IFN‐γ, which directly contribute to inflammatory processes (30, 31). Previous studies have indicated that alcohol exacerbates conditions like allergic dermatitis and allergic asthma by modulating autoimmune mediators (32, 33). Similarly, the EAP model reflects autoimmune-based inflammation. Consistent with clinical epidemiological findings (8), the incidence of CP/CPPS is notably higher among alcoholic drinkers. Additionally, alcohol is implicated in the regulation of prostatitis development. Our research further corroborated that alcohol exacerbates EAP severity, with feature of extensive inflammatory infiltration in the interstitium, resulting in significant pain and elevated levels of inflammatory cytokines.

The PI3K/AKT/mTOR network serves as a key regulator in many kinds of pathophysiological processes, such as inflammation, apoptosis (34, 35). Dysregulated PI3K/AKT/mTOR signaling is implicated in the initiation and modulation of autoimmunity and inflammation (36–38). Tang et al. demonstrated that lncRNA MEG3 inhibits PI3K/AKT/mTOR activation, suppressing inflammation in TNF-α-exposed psoriatic mice (39). Several drugs and their components are known to mitigate inflammation by targeting that axis (40–42). In our study, it was found that there exist obvious increase in p-PI3K, p-AKT, p-mTOR levels in EAP mice, which were further elevated following alcohol treatment. This finding suggests that alcohol exacerbates prostate inflammation by activating the inflammation-related that axis.

Several studies indicate infiltration of inflammatory cells like neutrophils, macrophages of CP/CPPS cases (10). Elevated Th1 cells contribute to pelvic pain and play a significant role in autoimmune prostatitis (22, 43). In EAP mice, prostate-infiltrating lymphocytes are predominantly Th1 cells, evidenced by upregulated cytoplasmic IFN-γ staining. Immunizing IL17A/F-deficient mice with prostate antigen induced a robust Th1 immune response, exacerbating persistent pelvic pain. This highlights the role of increased Th1 cell population in enhancing EAP susceptibility. The immune response involving Th1 cells and IFN-γ secretion is critical in CP/CPPS (44), suggesting a potential autoimmune mechanism targeting the prostate (45). Studies also report elevated IL-1β, TNF-α, IFN-γ levels in CP/CPPS patient seminal plasma (44), underscoring the importance of Th1 ratio in CP/CPPS development. However, the role of alcohol in regulating Th1 cells in CP/CPPS remains unclear. Here, using flow cytometry, we demonstrated that alcohol significantly increases both the quantity and proportion of Th1 cells. Moreover, LY294002, a PI3K suppressor, attenuated the PI3K/AKT/mTOR axis. Compared to EA mice, EA-LY mice showed reduced PI3K/AKT/mTOR pathway protein expression, fewer Th1 cells, and reduced prostate tissue inflammation. *In vitro* experiments with shRNA-PI3K-plasmid further supported these findings. Therefore, LY294002 represents a promising therapeutic approach for PI3K/AKT/mTOR network-related diseases such as CP/CPPS. LY294002 also holds potential for future research on the PI3K/AKT/mTOR network and associated diseases.

## 5 Conclusion

**Figure 6:**
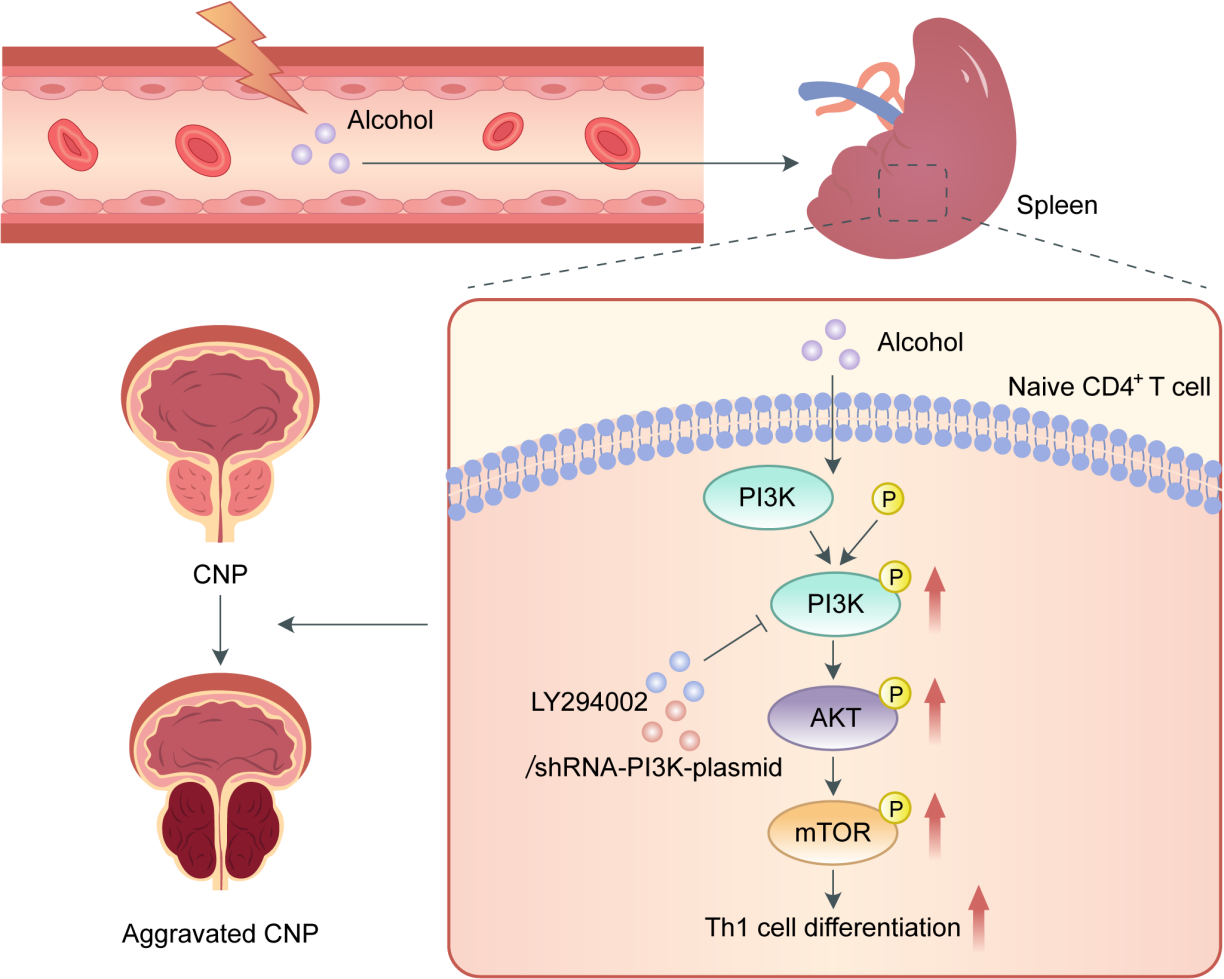
Schematic diagram of the pathogenic role of alcohol in EAP. In the process of EAP, alcohol activates the PI3K/AKT/mTOR pathway to increase the Th1 cell ratio, resulting in aggravation of EAP.

In summary, our study is the first to show that alcohol intake promotes Th1 cell differentiation and exacerbates EAP through activating the PI3K/AKT/mTOR pathway (**Figure 6**). Additionally, the role of LY294002 in inhibiting PI3K/AKT/mTOR pathway to relieve EAP indicated that it can served as a promising treatment target for CP/CPPS.

## Data Availability

The mass spectrometry proteomics data have been deposited to the ProteomeXchange Consortium via the PRIDE [1] partner repository with the dataset identifier PXD059384.

## References

[1] Liang C-Z Li H-J Wang Z-P Xing J-P Hu W-L Zhang T-F. The prevalence of prostatitis-like symptoms in China. J Urol. (2009) 182:558–63. doi: 10.1016/j.juro.2009.04.011 19524948

[2] Diri MA Gul M. Bipolar prostate thermotherapy for the improvement of chronic prostatitis symptoms and ejaculation problems. Aging Male. (2020) 23:1004–8. doi: 10.1080/13685538.2019.1650906 31397617

[3] Huang A Chang B Sun Y Lin H Li B Teng G. Disease spectrum of alcoholic liver disease in Beijing 302 Hospital from 2002 to 2013: A large tertiary referral hospital experience from 7422 patients. Med (Baltimore). (2017) 96:e6163. doi: 10.1097/MD.0000000000006163 PMC5319541 28207552

[4] Beresford TP Wongngamnit N Temple BA. Alcoholism: diagnosis, prognosis, epidemiology, and burden of the disease. Handb Clin Neurol. (2014) 125:3–13. doi: 10.1016/B978-0-444-62619-6.00001-X 25307565

[5] Bautista AP. Neutrophilic infiltration in alcoholic hepatitis. Alcohol. (2002) 27:17–21. doi: 10.1016/s0741-8329(02)00206-9 12062632

[6] Necro-inflammatory response of pancreatic acinar cells in the pathogenesis of acute alcoholic pancreatitis - PubMed . Available online at (Accessed September 2, 2024). 10.1038/cddis.2013.354 PMC3824664 24091659

[7] Abdul-Muneer PM Alikunju S Mishra V Schuetz H Szlachetka AM Burnham EL. Activation of NLRP3 inflammasome by cholesterol crystals in alcohol consumption induces atherosclerotic lesions. Brain Behav Immun. (2017) 62:291–305. doi: 10.1016/j.bbi.2017.02.014 28232172 PMC6378699

[8] Chen X Hu C Peng Y Lu J Yang NQ Chen L. Association of diet and lifestyle with chronic prostatitis/chronic pelvic pain syndrome and pain severity: a case-control study. Prostate Cancer Prostatic Dis. (2016) 19:92–9. doi: 10.1038/pcan.2015.57 26666410

[9] Zhang L-G Chen J Meng J-L Zhang Y Liu Y Zhan C-S. Effect of alcohol on chronic pelvic pain and prostatic inflammation in a mouse model of experimental autoimmune prostatitis. Prostate. (2019) 79:1439–49. doi: 10.1002/pros.23866 31233226

[10] Murphy SF Schaeffer AJ Thumbikat P. Immune mediators of chronic pelvic pain syndrome. Nat Rev Urol. (2014) 11:259–69. doi: 10.1038/nrurol.2014.63 PMC4986688 24686526

[11] Torres-Ruesta A Teo T-H Chan Y-H Rénia L Ng LFP. Pathogenic Th1 responses in CHIKV-induced inflammation and their modulation upon Plasmodium parasites co-infection. Immunol Rev. (2020) 294:80–91. doi: 10.1111/imr.12825 31773780 PMC7064921

[12] Samuel RO Ervolino E de Azevedo Queiroz ÍO Azuma MM Ferreira GT Cintra LTA. Th1/th2/th17/treg balance in apical periodontitis of normoglycemic and diabetic rats. J Endod. (2019) 45:1009–15. doi: 10.1016/j.joen.2019.05.003 31227229

[13] Jing H Gao X Xu L Lin H Zhang Z. H2S promotes a glycometabolism disorder by disturbing the Th1/Th2 balance during LPS-induced inflammation in the skeletal muscles of chickens. Chemosphere. (2019) 222:124–31. doi: 10.1016/j.chemosphere.2019.01.136 30703651

[14] Xiang K Wang B. Role of the PI3K−AKT−mTOR pathway in hepatitis B virus infection and replication. Mol Med Rep. (2018) 17:4713–9. doi: 10.3892/mmr.2018.8395 29328380

[15] Zhu Y Song D Song Y Wang X. Interferon gamma induces inflammatory responses through the interaction of CEACAM1 and PI3K in airway epithelial cells. J Transl Med. (2019) 17:147. doi: 10.1186/s12967-019-1894-3 31072323 PMC6507156

[16] Sun K Luo J Guo J Yao X Jing X Guo F. The PI3K/AKT/mTOR signaling pathway in osteoarthritis: a narrative review. Osteoarthritis Cartilage. (2020) 28:400–9. doi: 10.1016/j.joca.2020.02.027 32081707

[17] Zhang L-G Yu Z-Q Yang C Chen J Zhan C-S Chen X-G. Effect of Eriocalyxin B on prostatic inflammation and pelvic pain in a mouse model of experimental autoimmune prostatitis. Prostate. (2020) 80:1394–404. doi: 10.1002/pros.24065 32965686

[18] Zeng T Zhang C-L Song F-Y Zhao X-L Yu L-H Zhu Z-P. PI3K/Akt pathway activation was involved in acute ethanol-induced fatty liver in mice. Toxicology. (2012) 296:56–66. doi: 10.1016/j.tox.2012.03.005 22459179

[19] Huang X Li X Ma Q Xu Q Duan W Lei J. Retracted] Chronic alcohol exposure exacerbates inflammation and triggers pancreatic acinar−to−ductal metaplasia through PI3K/Akt/IKK. Int J Mol Med. (2023) 52:82. doi: 10.3892/ijmm.2023.5285 37503753 PMC10552770

[20] Rivero VE Cailleau C Depiante-Depaoli M Riera CM Carnaud C. Non-obese diabetic (NOD) mice are genetically susceptible to experimental autoimmune prostatitis (EAP). J Autoimmun. (1998) 11:603–10. doi: 10.1006/jaut.1998.0248 9878082

[21] Motrich RD Maccioni M Ponce AA Gatti GA Oberti JPM Rivero VE. Pathogenic consequences in semen quality of an autoimmune response against the prostate gland: from animal models to human disease. J Immunol. (2006) 177:957–67. doi: 10.4049/jimmunol.177.2.957 16818751

[22] Kurita M Yamaguchi H Okamoto K Kotera T Oka M. Chronic pelvic pain and prostate inflammation in rat experimental autoimmune prostatitis: Effect of a single treatment with phosphodiesterase 5 inhibitors on chronic pelvic pain. Prostate. (2018) 78:1157–65. doi: 10.1002/pros.23690 30009466

[23] Okamoto K Kurita M Yamaguchi H Numakura Y Oka M. Effect of tadalafil on chronic pelvic pain and prostatic inflammation in a rat model of experimental autoimmune prostatitis. Prostate. (2018) 78:707–13. doi: 10.1002/pros.23514 29577372

[24] Bertola A Mathews S Ki SH Wang H Gao B. Mouse model of chronic and binge ethanol feeding (the NIAAA model). Nat Protoc. (2013) 8:627–37. doi: 10.1038/nprot.2013.032 PMC3788579 23449255

[25] Shang X Lin K Yu R Zhu P Zhang Y Wang L. Resveratrol protects the myocardium in sepsis by activating the phosphatidylinositol 3-kinases (PI3K)/AKT/mammalian target of rapamycin (mTOR) pathway and inhibiting the nuclear factor-κB (NF-κB) signaling pathway. Med Sci Monit. (2019) 25:9290–8. doi: 10.12659/MSM.918369 PMC6911307 31806860

[26] Yin F Wu M Wei X Ren R Liu M Chen C. Hepatic NCoR1 deletion exacerbates alcohol-induced liver injury in mice by promoting CCL2-mediated monocyte-derived macrophage infiltration. Acta Pharmacol Sin. (2022) 43:2351–61. doi: 10.1038/s41401-022-00863-0 PMC9433401 35149852

[27] Lu S Jin H Nong T Li T Long K Chen Y. Hepatocyte-derived Fetuin-A promotes alcohol-associated liver disease in mice by inhibiting autophagy-lysosome degradation of TLR and M2 macrophage polarization. Free Radic Biol Med. (2024) 12:506–20. doi: 10.1016/j.freeradbiomed.2024.09.011 39277121

[28] Motrich RD Breser ML Molina RI Tissera A Olmedo JJ Rivero VE. Patients with chronic prostatitis/chronic pelvic pain syndrome show T helper type 1 (Th1) and Th17 self-reactive immune responses specific to prostate and seminal antigens and diminished semen quality. BJU Int. (2020) 126:379–87. doi: 10.1111/bju.15117 32437049

[29] Zhang M Liu Y Chen J Chen L Meng J Yang C. Single-cell multi-omics analysis presents the landscape of peripheral blood T-cell subsets in human chronic prostatitis/chronic pelvic pain syndrome. J Cell Mol Med. (2020) 24:14099–109. doi: 10.1111/jcmm.16021 PMC7754003 33124198

[30] López MC. Chronic alcohol consumption regulates the expression of poly immunoglobulin receptor (pIgR) and secretory IgA in the gut. Toxicol Appl Pharmacol. (2017) 333:84–91. doi: 10.1016/j.taap.2017.08.013 28843478

[31] Increased systemic and brain cytokine production and neuroinflammation by endotoxin following ethanol treatment - PubMed . Available online at (Accessed September 2, 2024). 10.1186/1742-2094-5-10 PMC2373291 18348728

[32] Sakazaki F Ogino H Arakawa T Okuno T Ueno H. Low-dose ethanol aggravates allergic dermatitis in mice. Alcohol. (2014) 48:501–8. doi: 10.1016/j.alcohol.2014.05.001 24953256

[33] Sisson JH. Alcohol and airways function in health and disease. Alcohol. (2007) 41:293–307. doi: 10.1016/j.alcohol.2007.06.003 17764883 PMC2081157

[34] Abdel-Aleem GA Khaleel EF Mostafa DG Elberier LK. Neuroprotective effect of resveratrol against brain ischemia reperfusion injury in rats entails reduction of DJ-1 protein expression and activation of PI3K/Akt/GSK3b survival pathway. Arch Physiol Biochem. (2016) 122:200–13. doi: 10.1080/13813455.2016.1182190 27109835

[35] Wang Z Ye Z Huang G Wang N Wang E Guo Q. Sevoflurane post-conditioning enhanced hippocampal neuron resistance to global cerebral ischemia induced by cardiac arrest in rats through PI3K/akt survival pathway. Front Cell Neurosci. (2016) 10:271. doi: 10.3389/fncel.2016.00271 27965539 PMC5127837

[36] Ghigo A Damilano F Braccini L Hirsch E. PI3K inhibition in inflammation: Toward tailored therapies for specific diseases. Bioessays. (2010) 32:185–96. doi: 10.1002/bies.200900150 20162662

[37] Malemud CJ. The PI3K/Akt/PTEN/mTOR pathway: a fruitful target for inducing cell death in rheumatoid arthritis? Future Med Chem. (2015) 7:1137–47. doi: 10.4155/fmc.15.55 26132523

[38] Li T-F Ma J Han X-W Jia Y-X Yuan H-F Shui S-F. Chrysin ameliorates cerebral ischemia/reperfusion (I/R) injury in rats by regulating the PI3K/Akt/mTOR pathway. Neurochem Int. (2019) 129:104496. doi: 10.1016/j.neuint.2019.104496 31247243

[39] Tang Z-L Zhang K Lv S-C Xu G-W Zhang J-F Jia H-Y. LncRNA MEG3 suppresses PI3K/AKT/mTOR signalling pathway to enhance autophagy and inhibit inflammation in TNF-α-treated keratinocytes and psoriatic mice. Cytokine. (2021) 148:155657. doi: 10.1016/j.cyto.2021.155657 34425525

[40] Fang Y Shi K Lu H Lu L Qiu B. Mingmu xiaomeng tablets restore autophagy and alleviate diabetic retinopathy by inhibiting PI3K/akt/mTOR signaling. Front Pharmacol. (2021) 12:632040. doi: 10.3389/fphar.2021.632040 33927618 PMC8077025

[41] Bao B Liu J Wan L Zhang Y Long Y Sun G. Xinfeng capsule inhibits immune inflammation in osteoarthritis by inhibiting the miR- 23a-3p/PETN/PI3K/AKT/mTOR pathway. Nan Fang Yi Ke Da Xue Xue Bao. (2021) 41:483–94. doi: 10.12122/j.issn.1673-4254.2021.04.02 PMC8110458 33963706

[42] Han G Zhang Y Li H. The combination treatment of curcumin and probucol protects chondrocytes from TNF-α Induced inflammation by enhancing autophagy and reducing apoptosis via the PI3K-akt-mTOR pathway. Oxid Med Cell Longev. (2021) 2021:5558066. doi: 10.1155/2021/5558066 34257809 PMC8249126

[43] Schaeffer EM. Re: Th1-Th17 cells contribute to the development of uropathogenic Escherichia coli-induced chronic pelvic pain. J Urol. (2014) 191:1808–9. doi: 10.1016/j.juro.2014.03.029 25280287

[44] Motrich RD Maccioni M Molina R Tissera A Olmedo J Riera CM. Presence of INFgamma-secreting lymphocytes specific to prostate antigens in a group of chronic prostatitis patients. Clin Immunol. (2005) 116:149–57. doi: 10.1016/j.clim.2005.03.011 15993362

[45] Penna G Amuchastegui S Cossetti C Aquilano F Mariani R Giarratana N. Spontaneous and prostatic steroid binding protein peptide-induced autoimmune prostatitis in the nonobese diabetic mouse. J Immunol. (2007) 179:1559–67. doi: 10.4049/jimmunol.179.3.1559 17641022

